# The Effect of Selected Factors on the Content of Fat-Soluble Vitamins and Macro-Elements in Raw Milk from Holstein-Friesian and Simmental Cows and Acid Curd Cheese (Tvarog)

**DOI:** 10.3390/ani10101800

**Published:** 2020-10-03

**Authors:** Jolanta Król, Agnieszka Wawryniuk, Aneta Brodziak, Joanna Barłowska, Beata Kuczyńska

**Affiliations:** 1 Institute of Quality Assessment and Processing of Animal Products, University of Life Sciences in Lublin, Akademicka 13, 20-950 Lublin, Poland; jolanta.krol@up.lublin.pl (J.K.); agn.wawryniuk@gmail.com (A.W.); joanna.barlowska@up.lublin.pl (J.B.); 2 Institute of Animal Sciences, Warsaw University of Life Sciences–SGGW, Nowoursynowska 166, 02-787 Warsaw, Poland; beata_kuczynska@sggw.edu.pl

**Keywords:** Holstein-Friesian breed, Simmental breed, milk, fat-soluble vitamins, macro-elements, retention rates, tvarog

## Abstract

**Simple Summary:**

Depending on the region, specific methods of cheese production are used. Acid coagulation is applied in the production of the traditional acid curd cheese highly valued in Poland, called ‘tvarog’. It is important to maintain the traditional nature of production that guarantees cheese with a high nutritional value and health-promoting properties. By conducting an analysis of tvarog, taking into account the quality of the raw milk and the type of starter cultures used, we found that the content of fat-soluble vitamins (A, D_3_, and E) mainly depended on the quality of the raw milk from which it was produced. A higher content of fat-soluble vitamins was found in milk obtained from Simmental cows kept in a traditional system in the spring/summer season and in the tvarog produced from it. Vitamin retention rates from raw milk to cheese were above 90%. In contrast, we noticed that the mineral composition of the cheese was not linked to the quality of the milk used. More Ca and Mg were lost with the whey, as indicated by the very low retention rates of these minerals from milk to tvarog (below 20%).

**Abstract:**

The study was conducted to determine the content and retention of selected fat-soluble vitamins and minerals in curd cheese–tvarog made by a traditional method, taking into account the effect of the quality of the raw milk and the type of starter cultures used. The raw milk used to make the tvarog was obtained in various conditions, i.e., with and without the use of pasture forage (in a traditional and an intensive system), in two production seasons (spring/summer and autumn/winter), from two breeds raised in Poland (the Black-and-White variety of Polish Holstein-Friesian and Simmental). Two variants of starter cultures were used to make tvarog: Freeze-dried DVS starters (Flora Danica) and pure cultures of mesophilic lactic acid bacteria. The acidity and content of protein, fat, selected fat-soluble vitamins (A, D_3_, and E), and selected macro-elements (Ca and Mg) were determined in samples of bulk milk and cheese. Retention rates of individual nutrients from the milk to the cheese were calculated. A higher content of fat-soluble vitamins was found in milk obtained from Simmental cows kept in a traditional system in the spring/summer season, as well as in the tvarog produced from it. Vitamin retention rates from the raw material to the tvarog were above 90%. The mineral composition of the cheese was not associated with the quality of the milk used. Very low retention rates from milk to cheese were obtained for Ca and Mg (below 20%). Higher retention rates were obtained in the spring/summer season when culture variant 1 was used. However, the starter culture was not found to significantly influence the concentration or retention of vitamins in the experimental cheese.

## 1. Introduction

Milk and dairy products are an important source of health-promoting substances in the human diet, including minerals and fat-soluble vitamins [1,2,3,4,5]. Vitamins are an important group of organic substances with high biological activity, essential for the growth and functioning of the body. They are involved in numerous vital processes, supporting metabolism and improving the activity of enzymes and catalyzing proteins. Together with β-carotene (provitamin A), vitamin A takes part in vision and reproduction, as well as in the differentiation, growth, and development of cells. Vitamin D plays a key role in calcium and phosphorus metabolism, conditioning normal bone mineralization, but also in immunological and anti-cancer processes. Vitamin E is one of the strongest antioxidants and inhibits cell ageing [4,6]. It should be emphasized that the human body only needs small amounts of vitamins, but their deficiency significantly affects health, and our endogenous metabolism is unable to produce them on its own, or produces them in negligible amounts. In the case of fat-soluble vitamins (A, D3, and E), milk and dairy products provide over 10% of vitamin A in the human diet and less than 10% of the others [3].

Among minerals, particular attention should be paid to calcium. Milk and dairy products are a rich source of calcium, supplying about 60% of its intake in the average diet. Calcium, primarily known as a building block for bones and teeth, has many other functions in the body: It takes part in the conductivity of nerve stimuli and the regulation of nerve excitability, muscle contractility, enzyme activation, and blood coagulation [4,7,8]. It should be emphasized that calcium absorption from dairy products reaches up to 45%, while its absorption from plant-based products usually does not exceed 10%. This is because dairy products contain substances that increase the bioavailability of calcium, such as vitamin D, phosphopeptides arising from the hydrolysis of casein, lactose, and basic amino acids. In the case of curd cheese, its low pH has an additional positive effect on the bioavailability of this element [7,9,10].

In Poland, the consumption of dairy products, including cheese, has been growing for several years. In 2019, the average annual consumption of this group of products was over 10 kg/person, including 5.40 kg of acid curd cheese (quark), referred to in Poland as ‘white cheese’ [11]. The high popularity of acid curd cheese in Poland stems from multi-generational tradition, long-established eating habits, and its high availability in a wide assortment and at a relatively low price. Poland and Russia are among the largest producers of non-ripened cheese, which, in Poland, is called ‘tvarog’. The range of dairy products available in other countries also includes non-ripened cheeses. Depending on the country, specific production methods are used, often associated with the region, and the products have different names [12]. In south-eastern Europe, white cheese in brine is popular [13]. Depending on the method used to coagulate milk proteins, we can distinguish acid-set curd cheeses and those produced using both acid and enzymes. Acid coagulation is used in the production of the traditional curd cheese highly valued in Poland. The specificity of the production technology of acid-set curd cheese consists of obtaining a curd from milk using pure starter cultures containing lactic acid bacteria, without coagulating enzymes or other additives [14,15].

The high nutritional value of the tvarog is mainly due to its content of complete animal protein, with very high digestibility, at a level of 96–97%, as well as its relatively low energy value. According to Barać et al. [13], curd cheese is a rich source of bioactive peptides. Moreover, it enriches the human diet with vitamins and minerals [7,15].

Research [5,16,17,18] indicates that the quality of raw milk is one of the main determinants of the value of dairy products, including curd cheese. Only high-quality raw milk with an appropriate chemical composition will result in a product with optimal nutritional value and health-promoting properties that fully meets consumer expectations [19]. The appropriate selection of starter cultures also significantly affects the production process, resulting in an attractive final product with favorable quality characteristics [15].

The aim of this study was to determine the content and retention of selected fat-soluble vitamins and minerals in curd cheese–tvarog made by the traditional method, taking into account the quality of the raw milk and the type of starter cultures used. The raw milk used to make the cheese was obtained in various conditions, i.e., with and without the use of pasture forage (in a traditional and an intensive system, in the spring/summer and autumn/winter seasons), from two breeds raised in Poland (Polish Holstein-Friesian and Simmental).

## 2. Materials and Methods

### 2.1. Materials

#### 2.1.1. Milk

The research material was bulk milk (24 samples in total) obtained from Polish Holstein-Friesian and Simmental cows. The study was carried out on three farms with Polish Holstein-Friesian and Simmental cows, including one using an intensive milk production system (group 1–12 milk samples were taken) and two using a traditional system (group 2–12 milk samples, i.e., six per farm). Cows from group 1 were kept in a free-stall barn, and the number of cows on the farm was 100. Two breeds were maintained on one farm. Milking parlor was used. The average daily milk yield per cow was 28.6 kg. The Partial Mixed Ratio (PMR) feeding system was used (year round consisting of bulk feeds, i.e., maize silage and haylage, and concentrate feed). The group 2 farms can be classified as low-input, with an average herd size of 15 cows kept in tie-stall barns. Farms were neighboring in one village, using the same feeding. A bucket milking system was used. The average daily milk yield per cow was 14.8 kg. Feeding in the spring and summer was mainly based on pasture forage, with the addition of hay and cereal meal, while the autumn and winter diet consisted of silage supplemented with hay and cereal meal.

All farms were subject to use value assessment for dairy cattle and met the requirements for milk production specified in Commission Regulation (EC) No. 1662/2006 of 6 November 2006 amending Regulation (EC) No. 853/2004 of the European Parliament and of the Council laying down specific hygiene rules for food of animal origin [20].

#### 2.1.2. Acid Curd Cheese–Tvarog

Before starting the production of tvarog, the raw material, i.e., non-standardized bulk milk, was heat-treated at 80 °C for 10 min in a water bath. Then, it was immediately cooled to 32–35 °C, and bacterial cultures were added (0.3 g/L). Two variants of starter cultures were used in the study: Starter variant 1—freeze-dried DVS starters—used to directly inoculate milk with lactic acid bacteria (Flora Danica by Chr Hansen, Graasten, Denmark), and starter variant 2—a traditional working starter obtained using freeze-dried inoculants of pure cultures of mesophilic lactic acid bacteria. Both variants contained *Lactococcus lactis* subsp. *cremoris*, *Lactococcus lactis* subsp. *lactis*, *Leuconostoc mesenteroides* subsp. *cremoris*, and *Lactococcus lactis* subsp. *diacetylactis*. The starters mainly differed in terms of the content of *Lactococcus lactis* subsp. *diacetylactis* strains, which are responsible for the synthesis of compounds determining flavor characteristics and CO_2_ production. The first inoculant contained 5–40% *Lactococcus lactis* subsp. *diacetylactis*, while the second contained 5–30%. The milk (1 L) was incubated at 30–32 °C for 12 h until a pH of about 4.6 was attained. The resulting curds were cut and briefly heated (to 45 °C), before being separated into drip bags made of cheese cloth. The whey was drained for about two hours. Tvarog was made under laboratory conditions three times in each of the two seasons. In total, 48 cheeses (24 cheeses on the basis of starter variant 1 and 24 on the basis of starter variant 2) were produced. The weight of each cheese was approx. 0.3 kg. All of the cheeses were made in the same conditions according to the same recipe.

### 2.2. Analyses

#### 2.2.1. Compositional and Microbiological Analysis of Milk and Cheese

The proximate chemical composition (protein, fat, and lactose) of the raw milk was determined with an Infrared Milk Analyser (Bentley Instruments, Chaska, MN, USA). To assess the hygienic quality of the raw milk, the total bacterial count at 30 °C [21] and somatic cell count (SCC) were determined with the Somacount 150 (Bentley Instruments, Chaska, MN, USA). The moisture content of cheese samples was determined using a drying method (at 102 °C), according to the Polish standard [22]. The protein content in the cheeses was analysed according to the Kjeldahl method, multiplying by a factor of 6.38 [23]. The fat content was assayed by the Weibull-Stoldt method, involving the acid hydrolysis (37% HCl, *w*/*w*) of lipids bound to proteins and carbohydrates, followed by hexane extraction. The analyses were carried out in triplicate.

#### 2.2.2. Determination of Fat-Soluble Vitamin Content in Milk, Whey, and Cheese

The concentrations of selected fat-soluble vitamins, i.e., A, D_3_, and E, in the milk, whey, and cheese, were determined by reversed-phase high performance liquid chromatography (RP-HPLC) using a ProStar Varian liquid chromatograph equipped with a fluorescence detector (Varian, Palo Alto, CA, USA). Samples were prepared by extracting fat according to the Röse-Gottlieb method, as modified by Hewavitharana et al. [24]. Compounds were separated on a Pursuit XRs 3-C18 column (Varian, Palo Alto, CA, USA) with a length of 150 mm and a diameter of 4.6 mm. The mobile phase was a mixture of acetonitrile, methanol, water, and dichloromethane (Sigma-Aldrich, Merck KGaA, Darmstadt, Germany), and the flow rate was set at 1 mL/min. Reference substances were analysed under identical conditions. Standard solutions of vitamins were used for this purpose: α-tocopherol (vitamin E) with ≥97% purity (HPLC), cholecalciferol (vitamin D_3_) with ≥98% purity (HPLC), and retinol (vitamin A) ≥99% (Sigma-Aldrich, Merck KGaA, Darmstadt, Germany). The qualitative identification of each substance was based on an analysis of retention times read from individual chromatograms using Star 6.2 Chromatography Workstation software (Varian, Palo Alto, CA, USA). Quantitative analysis was performed by the external standard method.

#### 2.2.3. Determination of the Ca and Mg Content in Milk, Whey, and Cheese

The content of Ca and Mg in the milk, whey, and cheese was determined by flame atomic absorption spectrometry (FAAS) with atomization in a strongly-oxidizing air-acetylene flame. The determinations were made using a Varian AA240FS Fast Sequential Atomic Absorption Spectrometer (Varian, Palo Alto, CA, USA). The test samples were prepared according to PN-EN 13804 [25]. Milk and whey in the amount of 1 mL each and about 0.5 g of cheese (within 0.0001 g) were collected in flasks, to which 1 mL 30% hydrochloric acid and 5 mL 65% suprapure nitric acid were added. The solutions were mineralized under an increased pressure in a CEM MARS 5 Xpress microwave digester (CEM Corporation, Matthews, NC, USA). Then, the mineralized samples were quantitatively transferred into 25 cm^3^ volumetric flasks using deionized water. The method was verified by testing the certified reference material NCS ZC 73015 Milk Powder NCS ZC in parallel with the test samples, in the same manner. The content of elements was read from a calibration curve of the dependency of the absorption on the content of the element. All analyses were performed in triplicate.

### 2.3. Calculation of the Degree of Retention

Based on the content of protein, fat, vitamins, and minerals in the milk and whey, the degree of retention of these substances in the experimental tvarog was calculated from the following equation [26]:

$$R\;(\%)=\frac{C_{milk}-C_{whey}}{C_{milk}}\times 100,$$

where C*_milk_* is the content of the component in milk (g) and C*_whey_* is the content of the component in whey (g).

### 2.4. Statistical Analysis

Statistical analysis of the results was performed using StatSoft Inc. Statistica ver. 13.1 (Dell, Round Rock, TX, USA), employing one-way and multi-way analysis of variance (ANOVA).

The following factors were included in the statistical analysis:

- The breed of cows (Polish Holstein-Friesian and Simmental) in the case of milk, whey, and tvarog;
- The production system (intensive and traditional) in the case of milk, whey, and tvarog;
- The production season (spring/summer and autumn/winter) in the case of milk, whey, and tvarog;
- The type of starter cultures used (freeze-dried DVS starters and traditional working starter) in the case of whey and tvarog.

The following effects were calculated:

- The breed of cows and the production system and season, as well as their interactions, on the basic chemical components, vitamin, and macro-element contents in bulk milk;
- The breed of cows, production system and season, and starter cultures on the amount of whey generated;
- The breeds of cows, production system and season, and starter cultures, as well as their interactions, on the basic chemical components, vitamin and macro-element contents, and retention rates in tvarog.

The applied one-way analysis of variance was analysed according to the following linear model:

Y_ij_ = µ + a_i_ + e_ij_

where Y_ij_ is the dependent variable (j-th observation from the i-th group), µ is the overall average effect, a_i_ is the effect of the i-th factor (group), and e_ij_ is the random error.

However, the applied multivariate analysis of variance was analysed according to the following linear model:

Y_ijklm_ = µ + a_i_ + b_j_ + c_k_ + d_l_ + (a_i_ × b_j_) + … + (a_i_ × b_j_ × c_k_ × d_l_) + e_ijklm_

where Y_ijklm_ is the dependent variable (m-th observation from the i-th group, … and l-th group); µ is the overall average effect; a_i_ is the effect of the i-th factor (group); b_j_ is the effect of the j-th factor; c_k_ is the effect of the k-th factor; d_l_ is the effect of the l-th factor; (a_i_ × b_j_), …, (a_i_ × b_j_ × c_k_ × d_l_) is the effect of the interaction of two or more factors; and e_ijklm_ is the random error.

The significance of differences between means for groups was determined by the Mann–Whitney test at *p* (α) = 0.05. The results are presented as means ($\bar{x}$) ± SD.

## 3. Results and Discussion

### 3.1. Milk

Milk employed for processing should have an appropriate acidity, indicating its freshness, with a pH value from 6.6 to 6.8 and titratable acidity of 6.0–7.5 °SH [27]. Lower pH values or a higher titratable acidity indicates overacidity. In our study, all milk samples met the requirements regarding the acidity standard. The raw milk was of a high hygienic quality, as indicated by the total microbial count (TMC) and somatic cell count (SCC). Neither of these qualitative parameters exceeded the admissible limit specified by regulations [20], i.e., 100,000/mL TMC and 400,000/mL SCC.

The cow breed, milk production system, and season were found to significantly influence most of the parameters analysed (Table 1). An important component of milk, determining its suitability for processing, is the total protein. Milk from Simmental cows had a significantly higher (*p* ≤ 0.05) protein content. Milk from Holstein-Friesian cows, on the other hand, had a significantly higher (*p* ≤ 0.01) content of fat. Milk obtained in the autumn/winter season from cows kept in an intensive system contained significantly more dry matter, including protein and fat. However, the analysed factors did not affect the lactose content, which varied from 4.68 to 4.76% (Table 1). According to Moynihan [28], the level of lactose and its ratio with casein significantly affected the lactic acid levels, which impacted the final pH, as well as the texture, functionality, and sensory properties of the cheeses. They concluded that controlling the lactose-to-casein ratio provides a way for cheesemakers to optimize the properties of Mozzarella cheese.

The presence of fat-soluble vitamins in milk is particularly important, partly due to their antioxidant properties. In our study, the production system had a statistically significant (*p* ≤ 0.01) effect on the concentration of all analysed vitamins. Milk obtained from farms using the traditional system had a higher content of vitamins (with a significantly lower fat content) compared to intensive farming (Table 1). The milk from the intensive production system contained about 30% less vitamin E, 25% less vitamin A, and 20% less vitamin D_3_. The content of fat-soluble vitamins in milk is therefore associated with the type of feed used. In the intensive system, one of the main factors responsible for the lower concentration of vitamins soluble in milk fat is the fact that cows are fed silage with a large amount of concentrate feed. In the traditional system, the use of grass silage or green forage resulted in a higher content of lipophilic vitamins. The results also indicate a significant (*p* ≤ 0.01) effect of the production season on the content of fat-soluble vitamins. Higher levels of these vitamins (by 40–45%) were found in the spring/summer season, when the animals grazed in the pasture. This indicates that pasture sward has higher levels of vitamin E and provitamin A than preserved feedstuffs, while the synthesis of vitamin D_3_ is induced by solar UV radiation. The increase in the content of fat-soluble vitamins in the milk of cows fed green forage has also been reported in other studies [1,2,29,30,31,32]. Importantly, the addition of concentrates to the diet of animals kept in the pasture has been shown to reduce the content of retinol in milk. The effect of the feeding system on the milk retinol concentration could be due to a difference in the retinol precursor concentration in the animals’ diet and/or a difference in the bioavailability of the precursors in the animal body. The grazing herbage positively influences the trans retinol level in milk [33].

Brodziak et al. [1] observed a relationship between the content of fat-soluble vitamins in milk and the cow housing system. In that study, a comparison of the bioactive status of the milk of Simmental cows kept in different housing systems (organic, traditional, and intensive) showed markedly better results for the organic and traditional systems. The concentration of vitamin A was 35% higher, vitamin D_3_ 23% higher, and vitamin E 90% higher than in the milk of cows from the intensive system. Butler et al. [2] showed a 50% increase in the amount of α-tocopherol in milk, which was due to the contribution of pasture forage in the cows’ diet. The influence of the production system on the concentration of vitamin E has also been reported by Kalac [30], who noted 0.38 mg of vitamin E in 100 g of milk fat in the milk of grass-fed cows and 0.21 mg in the milk of cows receiving maize silage. In contrast, Havemose et al. [34] found that, in the case of cows fed grass silage, the content of vitamin E (α-tocopherol) in the raw milk was 854 μg/L, while in the milk of cows fed maize silage, it was less than half that level (375 μg/L). Kuczyńska et al. [31] noted the most vitamin D_3_ in the milk of pasture-grazed cows, which have higher UV exposure than cows kept in barns during the winter.

An effect of the cow breed on the content of vitamins was only demonstrated for vitamin E (Table 1), whose content was significantly higher (*p* ≤ 0.01) in the milk of Simmental cows (1.71 mg/L; 41.20 µg/g fat) than in that of Holstein-Friesians (1.40 mg/L; 31.50 µg/g fat). The breed was not found to affect vitamin A and D_3_ levels in milk. Król et al. [35], in a study on the milk of cows of four breeds, i.e., Holstein-Friesian, Montbéliarde, Jersey, and Simmental, showed that the breed significantly affected the levels of fat-soluble vitamins. The milk of Holstein-Friesian cows, which provide the vast majority of milk in Poland, proved to be the poorest source of these substances, while the milk of Simmental cows contained the highest levels of vitamins A, E, and D_3_. Additionally, Ramalho et al. [36] indicated that Holstein cows produced milk less abundant in these vitamins than the autochthonous Portuguese breed Minhota.

Milk obtained from Simmental cows had a significantly (*p* ≤ 0.01) higher calcium content (1505.6 mg/L) than that of Holstein-Friesian cows (1249.4 mg/L). Irrespective of the breed of cows and production system, milk obtained in the autumn/winter season proved to be a richer source of calcium. Similarly, Król et al. [37], by assessing the content of minerals in milk produced on low-input farms, found a significantly higher concentration of this element in milk obtained in the autumn/winter season. The higher calcium content in the autumn/winter may result from the use of silage in the diet during this period. Zielińska et al. [38] showed that green forage had a slightly lower calcium content than silage made from it. According to the authors, this may be due to the metabolism of lactic acid bacteria. However, much of the calcium in milk is complexed to proteins [39], and when alterations in the milk protein occur, a significant decline in the amount of calcium/protein in the milk can be observed. The adverse effect of protein on calcium utilization is manifested when the ratio of the content of calcium to the content of protein in the diet (in dairy products) is less than 20. In the present study, the raw milk used for the production of curd cheese had high calcium-to-protein ratios ranging from 35.85 to 42.37 mg/g (Table 1), with the breed of cow and production system shown to have a significant effect on this parameter.

### 3.2. Whey

**Figure 1:**
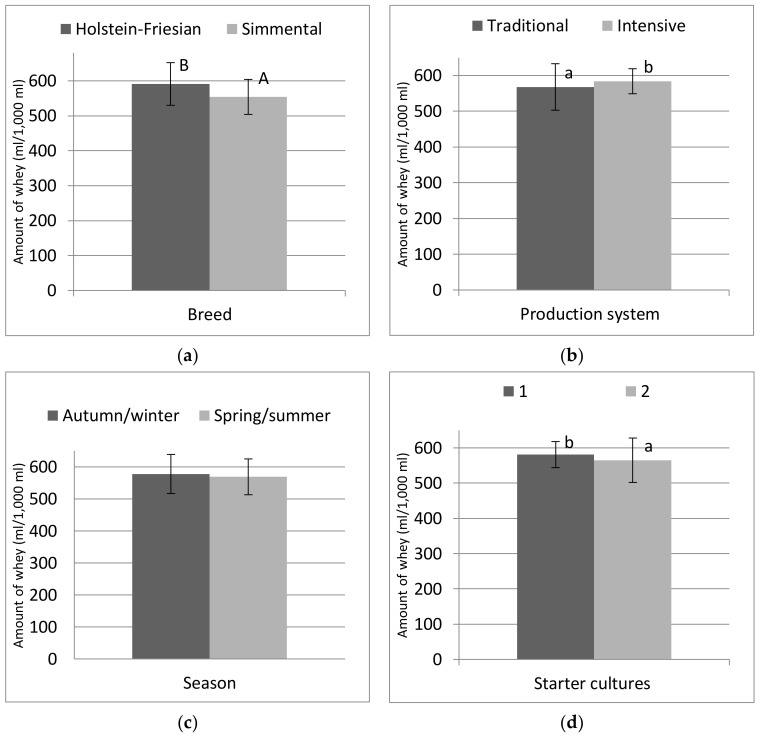
(**a**) The amount of whey generated, depending on the breed of cow, (**b**) milk production system, (**c**) season of production and (**d**) type of starter cultures (1—freeze-dried DVS starters; 2—traditional working starter). a, b, A, B—differences within the factor; a, b—significant at *p* ≤ 0.05; A, B—significant at *p* ≤ 0.01.

Whey is one of the most important by-products in the dairy industry, mainly generated in the production of cheese, including curd cheese. Analyses were performed on the whey produced during the production of cheese under laboratory conditions. Valuable components of the dry matter of milk were lost with the whey (about 50–60%). Tvarog produced from the milk of Simmental cows was characterized by a significantly (*p* ≤ 0.01) lower generation of whey, amounting to 554 mL, which is more advantageous for processing (Figure 1a). The amount of whey obtained from the milk of Holstein-Friesian cows was 7% higher. According to many authors [40,41,42], the addition of whey proteins reduces the separation of whey from the curd, which is linked to the ability of whey proteins to bind water. Therefore, the milk of Simmental cows probably contained more whey proteins than the milk of Holstein-Friesian cows. The level of lactose can also affect the amount of whey expelled [28], but no such relationship was observed in the present study. This was probably due the lack of differences in its content in the raw milk (Table 1). According to Dmytrów [43], the differences in the amount of whey generated can be explained by the specific characteristics of the bacteria in the starter culture, which cause varying degrees of acidification of the environment, thus determining the extent to which the casein curd shrinks and the amount of whey obtained. The starter cultures used in our study significantly (*p* ≤ 0.05) influenced the amount of whey obtained (Figure 1d). More whey was obtained in tvarog in which the Flora Danica inoculant was used (starter variant 1). Starter variant 2 was more advantageous in this respect, resulting in 3% less whey (Figure 1d). The curd formed using culture variant 1 was more susceptible to whey separation and leakage. An analysis of the impact of the production system showed that less whey was obtained in the production of cheese made from the milk of cows kept in the traditional system (Figure 1b). Significantly (*p* ≤ 0.05) more whey was obtained (about 3%) from tvarog produced from the milk of cows kept in an intensive system. The season, however, was not shown to significantly affect the amount of whey (Figure 1c). Skryplonek and Jasińska [41] obtained whey at the amount of up to 1/3 of the volume of raw milk used. The corresponding value in our study was over 50%. The content of fat-soluble vitamins and of Ca and Mg in the acid whey was only used to calculate their retention rates from the milk to the tvarog. It should be emphasized that the concentrations of these substances in whey mainly depended on the season in which the raw milk was procured. Their concentrations in whey were higher following the drainage of cheese made from milk obtained in the spring/summer season.

### 3.3. Acid Curd Cheese–Tvarog

The pH of tvarog was approx. 4.60. The content of dry matter components of the tvarog was directly linked to the quality of the milk used. A higher content of dry matter, including fat, was noted in the milk of Holstein-Friesian cows in the intensive system, and the cheese from this milk also contained more dry matter, including more fat (Table 2). The milk of Simmental cows was a richer source of protein, as was the cheese made from it. Irrespective of the breed of cow and the production system, a higher content of both protein and fat was noted in tvarog made from milk obtained in the autumn/winter season. The system and season of milk production and the breed of cows from which the milk was obtained were also shown to significantly affect the vitamin content in the cheese. The starter cultures, however, did not significantly influence the vitamin concentrations in the experimental cheese. Compared to the raw milk, tvarog proved to be a much richer source of fat-soluble vitamins. It contained several times more than the milk, with the greatest differences shown for vitamin A, whose content in the cheese was four times that noted in the milk. In addition, the cheese had three times as much vitamin D_3_ and vitamin E.

Irrespective of the breed of cows, production season, and starter cultures, a higher content of fat-soluble vitamins (A, D_3_, and E) was found in tvarog produced from the milk of cows raised in the traditional system (Table 3). Differences in vitamin content were 15–20% in favor of the traditional system in the autumn/winter season and 30–40% in spring/summer. The higher vitamin concentrations in the spring/summer may be linked to their higher content in the milk, which in turn is due to the contribution of pasture forage in the cows’ diet during this season. The cheese made from milk obtained in the pasture season contained about 20% more vitamin D_3_, 15% more vitamin E, and 10% more vitamin A, compared to the autumn/winter season. There were no significant seasonal differences in the intensive system, which is probably due to the cows’ diet, i.e., the use of the same feed throughout the year. The content of vitamins in the tvarog was also influenced by the breed of cows from which the milk was obtained, what can be associated with differences in the fat content. Cheese made from the milk of Simmental cows had a significantly higher content of vitamins D_3_ and E than cheese from the milk of Holstein-Friesians, as occurred with milk. The available literature lacks research specifically dealing with the content of fat-soluble vitamins in tvarog, and the few available studies concern ripened cheese. Lucas et al. [17], in a study on five French varieties of ripened farmhouse cheese, showed a significant effect (*p* < 0.001) of the milk composition on the composition of the cheese, indicating a significant linear correlation (*p* < 0.001) between the level of fat-soluble vitamins in the cheese and in the raw milk. It is worth noting that a higher content of vitamins A and E was found in Rocamadour cheese, produced by acid coagulation, compared to the other cheeses. As in our research, Revilla et al. [18] also reported that the milk production season had a significant impact on the concentration of vitamin A in both the milk and the ripened cheese produced from it. In the summer, the vitamin A content in this cheese was 212.79 μg/100 g fat, compared to 200.70 μg/100 g fat in the winter. The authors showed no effect of the season on the content of vitamin E. During ripening, they noted a decrease in the concentration of both vitamins in cheese. In their opinion, seasonal differences in retinol content may be due to different concentrations of the retinol precursor in the animals’ diet and/or their bioavailability. Hulshof et al. [44] also found that the vitamin A content in milk and dairy products in the Netherlands was about 20% higher in the summer than in the winter. Our analysis of the content of minerals in tvarog showed no effect of the cow breed, milk production system, season, or starter cultures on their content (Table 2). It should be noted, however, that tvarog proved to be a poorer source of minerals than the milk it was made from, due to substantial losses with the whey. The content of minerals in curd cheese therefore does not depend on their content in the milk used, which was also indicated in studies by Lucas et al. [17] and Manuelian et al. [5]. In their opinion, differences in the mineral composition of cheeses mainly result from the extent of acidification, and also partly from other technological factors (heating or salting). The Ca/protein and Mg/protein ratios also indicate a low content of minerals in curd cheese relative to raw milk (Table 2). The calcium-to-protein ratio in the cheese ranged from 7.10 to 13.35, while the ratio in the milk was 3–4 times higher. Similar relationships were found for the magnesium-to-protein ratio. Such low parameters are due to acidification of the raw milk to the isoelectric point. This has also been demonstrated by Siemianowski and Szpendowski [45]. According to Siemianowski et al. [46], a higher calcium content in curd cheese can be attained by using raw milk with a higher content of dry matter (more than 20%), which significantly prolongs the acidification time and thus reduces mineral losses during coagulation. This is due to the fact that in milk, minerals are present either in water-soluble form or in insoluble colloidal form bound to casein [17].

### 3.4. Milk-to-Cheese Retention

When milk is processed to make cheese, an effort is made to use the valuable nutrients of the raw milk to the greatest extent possible in the final product. An analysis of protein and fat retention from the raw milk to the finished product revealed significant differences (*p* < 0.05) for the season of milk production. Higher retention indices were obtained in the spring/summer: 87.2–88.6% for protein and 94.2–96.8% for fat (Table 4). The corresponding values for curd cheese made from milk obtained in the autumn/winter season were 85.6–87.9% for protein and 93.3–95.6% for fat. The other factors analysed were not found to affect the retention of protein or fat. Szpendowski et al. [47], by adding calcium chloride in the amount of 0.05% and pasteurizing the milk at 95 °C, attained 89.31% protein retention in the product. Increasing the concentration of calcium ions before heating the milk increases aggregation and interactions of whey proteins and the formation of complexes with casein [45]. The use of ultrafiltration in the production of curd cheese enables the utilization of up to 95% of milk proteins in the product [48]. The addition of transglutaminase to milk at the initial stage of treatment (before the starter cultures are introduced) also increases protein retention in the product [5].

The production system and season were found to significantly influence the retention of fat-soluble vitamins from the raw milk to the final product (Table 4). The breed only had a significant effect on the retention of vitamins D_3_ and E, while the starter cultures did not significantly affect vitamin retention rates. In all cases, the retention rate was above 90%. In the case of vitamin A retention, it is particularly worth noting the highly significant (*p* ≤ 0.01) effect of the season in which the milk for production of experimental cheese was obtained. Irrespective of the breed of cows, tvarog from the spring/summer retained more vitamin A. In the traditional system, the differences in favor of the spring/summer season ranged from 0.56 to 1.02 p.p. (percentage points), while in the case of the intensive system, they were lower, ranging from 0.06 to 0.72 p.p. Vitamin retention was significantly lower in the intensive system (*p* ≤ 0.05). Higher retention rates in tvarog made from the milk of cows housed in the traditional system were also found for vitamin D_3_ and E. The rates were lower in the intensive system (by 1 p.p. on average). The impact of the season on vitamin D_3_ and E retention was significant at *p* ≤ 0.05. The season influenced vitamin retention in favor of the spring/summer. The degree of vitamin D_3_ retention in the tvarog from the spring/summer season was about 1 p.p. higher than in cheese produced in the autumn/winter. Similar seasonal differences were found for vitamin E retention. An analysis of the effect of the cow breed (*p* ≤ 0.05) on vitamin D_3_ and E retention rates indicated that the retention rate of these vitamins was about 1 p.p. higher for tvarog produced from the milk of Simmental cows. We found no studies in the available literature on the retention of vitamins from raw milk to tvarog, but only on rennet-curd ripened cheese. Tippetts et al. [49] determined the retention of vitamin D_3_ in Cheddar cheese. In control cheese made from whole milk, they obtained vitamin D_3_ retention of 71.5%, but when they enriched the milk with this vitamin (in the form of an emulsion, using milk proteins as an emulsifier), the retention rate of vitamin D_3_ significantly increased to 96.6–97.4%. Stratulat et al. [50] presented similar research aimed at fortifying Cheddar cheese with antioxidant substances (vitamins A and E) using various encapsulation variants. The encapsulation of bioactive ingredients in cheese in the form of emulsified particles increased their retention in the curd, thereby maintaining their bioactivity and chemical stability during cheese storage. The authors obtained retention rates of 92% for vitamin E and 90% for vitamin A, compared to 66% and 75% for the control cheese. According to Hulshof et al. [44], retinol and ß-carotene retention in dairy products depends on the production technology used. In liquid and semi-liquid dairy products (pasteurized milk, buttermilk, and yogurt), the retention of both of these compounds is over 80% relative to the raw milk. They found lower retention rates for cheese, i.e., 50% for Gouda and 80% for Edam. During ripening, the retinol and ß-carotene content fell to about 30–40% relative to their content in milk. Lucas et al. [17], in their research on French varieties of ripened farmhouse cheese, showed losses of 34% for vitamin A and 67% for vitamin E during cheese production.

In contrast to the high retention rates of vitamins from milk to cheese, the values for minerals were very low, which indicates high losses with the whey. Ca retention ranged from 18.12% to 26.35%, and Mg retention from 16.58% to 28.44%. It should be noted that the production season, as well as the starter cultures, significantly influenced the level of retention. Higher retention rates, irrespective of the breed of cows and the system of milk production, were noted for cheese made from milk obtained in the spring/summer season using starter variant 1. Cheese from milk obtained in the spring/summer retained about 5 p.p. more Ca and Mg than autumn/winter cheese. Such large losses of minerals in cheese production are consistent with the results reported by other authors [5,17,51,52]. Baran et al. [51] obtained calcium retention rates of only 15–18% in acid cheese. The authors reported much higher rates for acid-rennet cheese (58–85%). This indicates that the higher the acidity of the milk, the greater the loss of minerals with the whey, including Ca and Mg. Reducing the pH of milk during coagulation causes the gradual solubilization of colloidal calcium phosphate, as well as magnesium bound with casein [17]. Jana and Mandal [53] found that a decrease in pH (from 6.2 to 5.3) in the production of Mozzarella cheese led to a significantly lower content of minerals in the cheese.

## 4. Conclusions

In sum, the quality of curd cheese is closely linked to the quality of the milk it is made from. The most decisive factor determining this is the production system. In the traditional system, the season of production proved to be significant as well, due to differences in the diet, i.e., the use of pasture forage in the summer. Differences in the content of basic nutrients and vitamins were observed in both the raw milk and the curd cheeses. The starter cultures, however, were not found to significantly affect vitamin concentrations in the experimental cheese. The high retention rates (above 90%) obtained also indicated the high health-promoting value of tvarog. In contrast, the mineral composition of the cheese was not linked to the quality of the milk used. It should be noted that more Ca and Mg were lost with the whey, as indicated by the very low retention rates of these minerals from milk to tvarog (below 20%). Therefore, it can be concluded that cheeses produced from milk of Simmental cows obtained during the pasture period are a distinctive product. The production of tvarog based on the milk of Simmental cows should be promoted, especially on small-scale traditional farms, where husbandry is often based on local breeds.

## Figures and Tables

**Table 1:** Content of basic chemical components, selected fat-soluble vitamins, and macro-elements in bulk milk, depending on the experimental factors ($\bar{x}$ ± SD).

Traits	Breed		System		Season		Interactions *p*-Value			
	Holstein-Friesian (HF)	Simmental (S)	Traditional (T)	Intensive (I)	Autumn/Winter	Spring/Summer	Breed × System	Breed × Season	System × Season	Breed × System × Season
Dry matter (%)	13.15 ^B^ ± 0.72	12.86 ^A^ ± 0.68	12.67 ^A^ ± 0.64	13.48 ^B^ ± 1.18	13.53 ^B^ ± 0.82	12.68 ^A^ ± 0.71	0.028	0.044	0.014	0.385
Lactose (%)	4.76 ± 0.78	4.71 ± 0.32	4.75 ± 0.54	4.68 ± 0.38	4.73 ± 0.41	4.75 ± 0.65	0.438	0.226	0.557	0.628
Protein (%)	3.45 ^a^ ± 0.25	3.57 ^b^ ± 0.19	3.45 ^A^ ± 0.21	3.72 ^B^ ± 0.08	3.61 ^B^ ± 0.18	3.45 ^A^ ± 0.25	0.032	0.014	0.008	0.190
Fat (%)	4.40 ^B^ ± 0.32	4.15 ^A^ ± 0.24	4.06 ^a^ ± 0.32	4.42 ^b^ ± 0.20	4.43 ^b^ ± 0.31	4.13 ^a^ ± 0.32	0.020	0.038	0.025	0.491
Vitamin A (mg/L)	0.37 ± 0.10	0.40 ± 0.10	0.41 ^B^ ± 0.10	0.32 ^A^ ± 0.04	0.33 ^A^ ± 0.06	0.46 ^B^ ± 0.10	0.310	0.628	0.001	0.273
Vitamin A (μg/g fat)	8.86 ± 1.17	9.64 ± 2.22	10.10 ^B^ ± 2.46	7.24 ^A^ ± 0.73	7.45 ^A^ ± 1.99	11.14 ^B^ ± 2.67	0.241	0.372	0.008	0.155
Vitamin D_3_ (μg/L)	0.56 ± 0.15	0.59 ± 0.15	0.61 ^B^ ± 0.16	0.50 ^A^ ± 0.05	0.48 ^A^ ± 0.08	0.70 ^B^ ± 0.14	0.610	0.733	0.000	0.661
Vitamin D_3_ (μg/g fat)	0.013 ± 0.002	0.014 ± 0.004	0.015 ^B^ ± 0.001	0.011 ^A^ ± 0.002	0.011 ^A^ ± 0.002	0.017 ^B^ ± 0.004	0.400	0.339	0.005	0.220
Vitamin E (mg/L)	1.40 ^A^ ± 0.33	1.71 ^B^ ± 0.56	1.69 ^B^ ± 0.49	1.21 ^A^ ± 0.11	1.32 ^A^ ± 0.13	1.87 ^B^ ± 0.59	0.193	0.182	0.002	0.197
Vitamin E (μg/g fat)	31.50 ^A^ ± 2.34	41.20 ^B^ ± 3.46	48.27 ^B^ ± 4.05	27.38 ^A^ ± 1.13	29.80 ^A^ ± 1.37	45.28 ^B^ ± 2.98	0.285	0.307	0.010	0.334
Ca (mg/L)	1249.4 ^A^ ± 156.5	1505.6 ^B^ ± 258.2	1404.7 ± 178.5	1388.2 ± 256.5	1534.4 ^B^ ± 291.5	1235.2 ^A^ ± 17.1	0.034	0.016	0.182	0.099
Mg (mg/L)	119.4 ± 24.6	112.3 ± 21.3	118.5 ± 24.6	114.5 ± 18.6	126.4 ± 18.4	104.6 ± 20.5	0.085	0.030	0.218	0.312
Ca:protein (mg/g)	36.41^A^ ± 3.23	41.98 ^B^ ± 3.74	39.97 ± 3.44	37.40 ± 3.18	42.37 ^B^ ± 4.02	35.85 ^A^ ± 3.11	0.048	0.012	0.438	0.603
Mg:protein (mg/g)	3.46 ± 0.24	3.15 ± 0.19	3.45 ± 0.32	3.12 ± 0.21	3.54 ± 0.31	3.06 ± 0.28	0.102	0.051	0.297	0.491

SD—standard deviation; Ca—calcium; Mg—magnesium. ^a^, ^b^, ^A^, ^B^—statistically significant differences within a given factor: ^a^, ^b^—at *p* ≤ 0.05; ^A^, ^B^—at *p* ≤ 0.01.

**Table 2:** Content of the basic chemical components and selected macro-elements in acid curd cheese (tvarog), depending on the experimental factors ($\bar{x}$ ± SD).

Traits	Season	Traditional System				Intensive System				Effect of Factor (*p*-Value)			
		Milk from Simmental Cows		Milk from Holstein-Friesian Cows		Milk from Simmental Cows		Milk from Holstein-Friesian Cows					
		Starter Cultures *								Breed	System	Season	Culture
		1	2	1	2	1	2	1	2				
Moisture (%)	Autumn/winter	74.44 ± 4.31	70.62 ± 3.29	69.50 ± 3.04	68.67 ± 1.49	70.81 ± 1.64	69.68 ± 3.81	64.04 ± 4.28	62.88 ± 3.74	0.009	0.043	0.031	0.020
	Spring/summer	75.17 ± 2.43	73.18 ± 4.27	74.33 ± 2.38	73.64 ± 2.65	74.64 ± 1.26	70.20 ± 5.16	71.04 ± 2.12	69.94 ± 1.83				
Protein (%)	Autumn/winter	13.33 ± 1.34	14.37 ± 1.60	11.85 ± 0.87	13.20 ± 0.65	18.83 ± 0.57	20.24 ± 0.37	15.04 ± 0.10	15.15 ± 0.52	0.047	0.000	0.000	0.000
	Spring/summer	9.73 ± 1.04	11.44 ± 1.58	9.38 ± 1.15	12.82 ± 3.01	16.02 ± 0.64	17.39 ± 0.27	13.77 ± 0.28	13.90 ± 0.92				
Fat, dry matter (%)	Autumn/winter	36.75 ± 1.83	40.71 ± 5.72	40.40 ± 1.23	40.85 ± 2.54	39.28 ± 1.86	42.06 ± 3.84	47.08 ± 3.9	46.83 ± 3.76	0.000	0.000	0.028	0.113
	Spring/summer	35.31 ± 5.19	38.43 ± 1.18	38.86 ± 3.84	40.31 ± 4.55	36.10 ± 1.17	39.60 ± 1.89	43.12 ± 1.87	44.50 ± 2.35				
Ca (mg/kg)	Autumn/winter	1376.8 ± 158.3	1425.8 ± 178.3	1310.9 ± 217.5	1382.5 ± 258.3	1338.4 ± 198.7	1389.2 ± 221.6	1286.6 ± 287.3	1335.4 ± 213.8	0.093	0.144	0.038	0.043
	Spring/summer	1289.2 ± 282.4	1334.5 ± 235.3	1249.4 ± 197.6	1298.0 ± 235.7	1317.2 ± 236.8	1356.5 ± 303.5	1274.2 ± 245.8	1318.3 ± 253.6				
Mg (mg/kg)	Autumn/winter	113.5 ± 15.3	116.4 ± 18.3	119.6 ± 17.4	121.0 ± 21.3	115.3 ± 13.9	116.6 ± 16.2	122.2 ± 20.4	122.9 ± 19.4	0.278	0.734	0.445	0.581
	Spring/summer	106.8 ± 11.4	107.23 ± 19.3	112.7 ± 10.9	113.0 ± 13.6	112.7 ± 17.4	115.8 ± 20.3	119.3 ± 17.8	120.1 ± 19.3				
Ca:protein (mg/g)	Autumn/winter	10.50 ± 1.02	10.11 ± 0.98	10.83 ± 1.34	10.51 ± 1.42	7.28 ± 0.64	7.10 ± 0.58	8.94 ± 0.62	9.05 ± 0.84	0.315	0.043	0.038	0.109
	Spring/summer	13.26 ± 1.12	11.88 ± 1.03	13.35 ± 1.22	10.26 ± 0.87	8.30 ± 0.66	8.21 ± 0.72	9.42 ± 0.69	9.73 ± 0.73				
Mg:protein (mg/g)	Autumn/winter	0.85 ± 0.10	0.81 ± 0.08	1.03 ± 0.11	0.96 ± 0.07	0.64 ± 0.08	0.59 ± 0.04	0.83 ± 0.07	0.84 ± 0.08	0.037	0.063	0.048	0.262
	Spring/summer	1.14 ± 0.11	0.96 ± 0.10	1.28 ± 0.14	0.91 ± 0.08	0.74 ± 0.05	0.69 ± 0.06	0.81 ± 0.09	0.82 ± 0.06				

SD—standard deviation; Ca—calcium; Mg—magnesium. * 1—freeze-dried DVS starters; 2—traditional working starter.

**Table 3:** Content of selected fat-soluble vitamins in acid curd cheese (tvarog), depending on the experimental factors ($\bar{x}$ ± SD).

Traits	Season	Traditional System				Intensive System				Effect of Factor (*p*-Value)			
		Milk from Simmental Cows		Milk from Holstein-Friesian Cows		Milk from Simmental Cows		Milk from Holstein-Friesian Cows					
		Starter Cultures *								Breed	System	Season	Culture
		1	2	1	2	1	2	1	2				
Vitamin A (mg/kg)	Autumn/winter	1.39 ± 0.01	1.40 ± 0.01	1.37 ± 0.02	1.35 ± 0.15	1.14 ± 0.13	1.31 ± 0.31	1.09 ± 0.07	1.03 ± 0.06	0.048	0.018	0.036	0.226
	Spring/summer	1.55 ± 0.05	1.56 ± 0.024	1.47 ± 0.037	1.46 ± 0.027	1.16 ± 0.08	1.20 ± 0.05	1.11 ± 0.08	1.106 ± 0.07				
Vitamin A (μg/g fat)	Autumn/winter	15.05 ± 1.96	11.69 ± 1.06	11.8 ± 0.96	10.74 ± 1.20	10.13 ± 0.89	10.39 ± 1.36	6.48 ± 0.72	5.83 ± 0.52	0.042	0.025	0.047	0.341
	Spring/summer	16.74 ± 1.34	13.02 ± 1.42	12.28 ± 1.35	11.64 ± 1.42	10.32 ± 1.15	9.61 ± 0.97	6.64 ± 0.80	6.23 ± 0.61				
Vitamin D_3_ (μg/kg)	Autumn/winter	1.74 ± 0.02	1.76 ± 0.04	1.65 ± 0.03	1.64 ± 0.03	1.48 ± 0.11	1.49 ± 0.05	1.408 ± 0.09	1.400 ± 0.11	0.023	0.031	0.027	0.151
	Spring/summer	2.19 ± 0.03	2.27 ± 0.25	1.85 ± 0.09	1.93 ± 0.06	1.51 ± 0.09	1.48 ± 0.01	1.42 ± 0.07	1.416 ± 0.05				
Vitamin D_3_ (μg/g fat)	Autumn/winter	0.018 ± 0.002	0.015 ± 0.003	0.014 ± 0.004	0.013 ± 0.003	0.013 ± 0.003	0.012 ± 0.002	0.008 ± 0.001	0.008 ± 0.001	0.036	0.027	0.028	0.267
	Spring/summer	0.024 ± 0.005	0.020 ± 0.004	0.016 ± 0.003	0.016 ± 0.002	0.014 ± 0.004	0.012 ± 0.003	0.008 ± 0.002	0.008 ± 0.001				
Vitamin E (mg/kg)	Autumn/winter	3.67 ± 0.02	3.71 ± 0.06	3.29 ± 0.02	3.29 ± 0.01	3.01 ± 0.13	3.19 ± 0.28	2.99 ± 0.09	2.84 ± 0.11	0.017	0.040	0.023	0.708
	Spring/summer	4.09 ± 0.03	4.09 ± 0.02	3.88 ± 0.03	3.89 ± 0.02	3.19 ± 0.17	3.18 ± 0.12	3.02 ± 0.04	2.95 ± 0.17				
Vitamin E (μg/g fat)	Autumn/winter	39.72 ± 4.68	31.03 ± 4.02	27.48 ± 3.09	26.21 ± 4.01	26.73 ± 3.17	25.34 ± 3.07	17.8 ± 2.21	16.06 ± 2.04	0.012	0.048	0.027	0.542
	Spring/summer	44.23 ± 6.21	34.12 ± 3.75	32.31 ± 3.33	30.92 ± 4.20	28.26 ± 3.05	25.31 ± 2.99	17.89 ± 1.86	16.65 ± 1.67				

SD—standard deviation; Ca—calcium; Mg—magnesium. * 1—freeze-dried DVS starters; 2—traditional working starter.

**Table 4:** Retention rates of protein, fat, selected fat-soluble vitamins, and macro-elements from raw milk to curd cheese, depending on the experimental factors ($\bar{x}$ ± SD).

Retention Rate (%)	Season	Traditional System				Intensive System				Effect of Factor *p*-Value			
		Milk from Simmental Cows		Milk from Holstein-Friesian Cows		Milk from Simmental Cows		Milk from Holstein-Friesian Cows					
		Starter Cultures *								Breed	System	Season	Culture
		1	2	1	2	1	2	1	2				
Protein	Autumn/winter	85.9 ± 1.1	86.3 ± 0.7	85.6 ± 0.8	85.9 ± 1.1	87.2 ± 0.9	87.9 ± 1.1	86.3 ± 0.7	86.6 ± 1.0	0.649	0.341	0.022	0.130
	Spring/summer	87.2 ± 0.9	88.0 ± 0.9	87.4 ± 1.0	87.8 ± 0.9	88.3 ± 0.8	88.6 ± 2.0	87.9 ± 1.2	88.1 ± 0.9				
Fat	Autumn/winter	93.3 ± 4.4	94.0 ± 2.4	93.6 ± 3.8	94.0 ± 3.7	95.6 ± 2.7	95.8 ± 3.4	94.1 ± 3.1	95.5 ± 1.3	0.547	0.187	0.041	0.740
	Spring/summer	94.2 ± 1.7	96.2 ± 0.6	95.5 ± 2.8	96.1 ± 3.2	94.8 ± 2.3	96.2 ± 1.5	96.8 ± 2.9	96.0 ± 1.8				
Vitamin A	Autumn/winter	93.7 ± 1.2	93.9 ± 1.1	93.3 ± 1.3	93.9 ± 0.9	93.3 ± 1.0	93.2 ± 2.1	92.4 ± 1.4	93.2 ± 0.8	0.060	0.032	0.008	0.488
	Spring/summer	94.5 ± 0.6	95.4 ± 1.2	94.2 ± 0.8	94.2 ± 0.8	94.4 ± 2.1	94.9 ± 1.2	93.7 ± 2.1	94.3 ± 1.4				
Vitamin D_3_	Autumn/winter	94.1 ± 0.3	94.4 ± 0.2	93.3 ± 0.3	93.8 ± 0.3	93.2 ± 0.3	93.5 ± 0.1	92.6 ± 0.1	92.7 ± 0.1	0.018	0.026	0.002	0.896
	Spring/summer	95.3 ± 0.2	95.6 ± 0.4	94.0 ± 0.5	94.0 ± 0.1	92.6 ± 0.8	92.3 ± 1.0	91.8 ± 1.1	91.7 ± 2.1				
Vitamin E	Autumn/winter	93.5 ± 0.3	93.6 ± 0.3	92.2 ± 0.6	92.5 ± 0.5	93.3 ± 0.5	92.9 ± 0.1	92.1 ± 0.3	91.2 ± 0.2	0.006	0.036	0.050	0.287
	Spring/summer	94.4 ± 0.3	94.7 ± 0.2	92.7 ± 0.3	93.0 ± 0.3	92.3 ± 0.2	92.6 ± 0.9	90.5 ± 1.1	91.1 ± 0.5				
Ca	Autumn/winter	20.1 ± 1.9	18.1 ± 1.8	20.3 ± 2.0	20.1 ± 2.1	19.8 ± 1.8	18.6 ± 1.6	20.8 ± 2.0	20.2 ± 1.9	0.228	0.361	0.015	0.037
	Spring/summer	24.4 ± 1.2	21.6 ± 1.5	26.4 ± 2.1	25.1 ± 1.7	23.8 ± 1.5	22.1 ± 1.8	25.9 ± 2.0	24.8 ± 2.0				
Mg	Autumn/winter	18.6 ± 1.0	16.6 ± 1.3	22.0 ± 1.7	19.1 ± 1.2	19.3 ± 2.1	16.9 ± 1.6	21.4 ± 1.9	18.4 ± 1.2	0.089	0.314	0.003	0.019
	Spring/Summer	26.1 ± 1.2	23.1 ± 1.1	28.4 ± 2.0	24.2 ± 1.6	24.6 ± 1.9	20.3 ± 1.2	24.9 ± 1.5	20.5 ± 1.4				

SD—standard deviation; Ca—calcium; Mg—magnesium. * 1—freeze-dried DVS starters; 2—traditional working starter.
